# Impact of type and dose of oral polyunsaturated fatty acid supplementation on disease activity in inflammatory rheumatic diseases: a systematic literature review and meta-analysis

**DOI:** 10.1186/s13075-022-02781-2

**Published:** 2022-05-07

**Authors:** Johanna Sigaux, Sylvain Mathieu, Yann Nguyen, Pauline Sanchez, Jean-Guillaume Letarouilly, Martin Soubrier, Sébastien Czernichow, René-Marc Flipo, Jérémie Sellam, Claire Daïen

**Affiliations:** 1 Department of Rheumatology, Hôpital Avicenne, APHP, INSERM U1125, Université Sorbonne Paris Nord, 125 rue de Stalingrad, 93000 Bobigny, France; 2 Department of Rheumatology, CHU Gabriel-Montpied, Clermont-Ferrand, France; 3 Department of Internal Medicine, Hôpital Beaujon, APHP Nord, Université de Paris, Clichy, France; 4 Department of Rheumatology, CHU de Montpellier, University of Montpellier, PhyMedExp, INSERM, CNRS UMR, Montpellier, France; 5 Department of Rheumatology, CHU Lille, Université de Lille, Lille, France; 6 Department of Nutrition, Specialized Obesity Center, Hôpital Européen Georges Pompidou, Université de Paris, APHP, Paris, France; 7 Epidemiology and Biostatistics Sorbonne Paris City Center, UMR1153, Institut National de la Santé et de la Recherche Médicale, Paris, France; 8 DMU 3ID, Hôpital Saint Antoine, APHP, CRSA Inserm UMRS_938, Sorbonne Université, Paris, France

**Keywords:** Inflammatory rheumatic diseases, Rheumatoid arthritis, Polyunsaturated fatty acids, Omega-3, Omega-6, Meta-analysis, Systematic literature review

## Abstract

**Background:**

Polyunsaturated fatty acid (PUFA) supplementation has been reported to improve disease activity in inflammatory rheumatic diseases (IRDs). However, data are often conflicting and studies insufficiently large to draw conclusions. This systematic literature review and meta-analysis aimed to better estimate the effect of oral supplementation with omega (n)-3 and n-6 PUFA on IRD activity in terms of duration, dose, type, and source.

**Methods:**

The literature was searched in PubMed, EMBASE, and Cochrane Library databases up to October 2020. Studies were reviewed in accordance with PRISMA guidelines. The effect of PUFA supplementation on disease activity was expressed as the standardized mean difference (95% CI). Metaregression and subgroup analyses involved type of IRD, Jadad score, PUFA source (animal or vegetable), and doses.

**Results:**

We obtained 42 references; 30 randomized controlled studies were included comparing the effects of PUFA versus control on disease activity (710 IRD patients receiving PUFA supplementation and 710 controls, most with rheumatoid arthritis). We found a significant improvement in pain, swollen and tender joint count, Disease Activity Score in 28 joints, and Health Assessment Questionnaire score in IRD patients receiving PUFA supplementation as compared with controls, with a significant decrease in erythrocyte sedimentation rate but not C-reactive protein level. Although meta-regression revealed no difference by IRD type or source or dose of PUFA supplementation, subgroup analysis revealed more parameters significantly improved with animal- than vegetable-derived PUFAs and 3- to 6-month supplementation. Most studies examined high-dose supplementation (>2 g/day).

**Conclusion:**

PUFA consumption, especially omega-3 from animal source >2 g/day, may improve IRD activity and might be an adjuvant therapy in rheumatoid arthritis.

**Trial registration:**

The protocol was registered at PROSPERO (CRD42021253685).

**Supplementary Information:**

The online version contains supplementary material available at 10.1186/s13075-022-02781-2.

## Background

In the last 20 years, knowledge about rheumatoid arthritis (RA) and other inflammatory rheumatic diseases (IRDs) has greatly improved and led to effective treatments. Nevertheless, patients’ unmet needs remain substantial, and a growing number of patients require alternative therapies as a complement to traditional biological and non-biological disease-modifying anti-rheumatic drugs. Diet is now considered a major component of chronic diseases, especially in RA [1]. Nutrition plays a role in the emergence of these diseases [2] as well as their evolution and activity [3, 4]. Nevertheless, studying the potential effects of diet remains a great challenge because of the complexity and variety of the components, and studies are often disparate in quality and methods. Unlike trials of Mediterranean, vegan, and gluten-free diets, randomized controlled trials (RCTs) of supplementation with n-3 polyunsaturated fatty acids (n-3 PUFAs) in patients with IRD, especially encapsulated oil, have been of high quality. Therefore, studies of n-3 PUFA supplementation, mostly derived from fish oil, are the most numerous and are also supported by a pathophysiological rationale, because n-3 PUFAs have anti-inflammatory effects [5], promote immune-regulation activities [6], and modulate gut microbiota and gut permeability [7].

Little is known about the effect of n-6 PUFAs. N-6 PUFAs are considered pro-inflammatory components because linoleic acid (LA), included in more than 50% of the most-consumed vegetable oils, is a precursor of arachidonic acid, itself a precursor of inflammatory mediators [8]. Nevertheless, some n-6 PUFAs may have beneficial effects on health. For example, gamma-linolenic acid (GLA), found in certain oils such as evening primrose oil, may have an anti-inflammatory action due to the production of prostaglandin E_1_ and other eicosanoids [9]. Moreover, it has been shown that GLA supplementation decreases ex vivo production of leukotriene B_4_ by neutrophils [10]. However, few RCTs have investigated the effect of n-6 PUFA supplementation on IRD activity.

Five heterogenous meta-analyses investigating the effect of n-3 supplementation on disease activity have been published since 1995 [11–15]. Three included exclusively RA patients [11, 13, 15] and one, published in 2012, focused on oral n-3 PUFA supplementation> 2.7 g/day [13]. The results are broadly consistent with a beneficial effect of n-3 PUFA supplementation on tender joint count (TJC), pain, morning stiffness (MS), and non-steroidal anti-inflammatory drug (NSAID) use. Conversely, the effects on swollen joint count (SJC), inflammatory biomarkers, Health Assessment Questionnaire (HAQ) score, and Disease Activity Score in 28 joints (DAS28) are still equivocal.

We performed an updated meta-analysis and meta-regression (by type of IRD, Jadad quality score, PUFA source [animal or vegetable], and dose) of published studies or abstracts. We aimed to update previous meta-analyses with recent literature and better estimate the effect of oral n-3 and n-6 PUFA supplementation by dose and source on disease activity and pain in patients with IRD. This work was conducted to inform the recommendations of the French Society of Rheumatology on dietary practices in IRD [16].

## Methods

A systematic review of the published literature was performed in accordance with the Preferred Reporting Items for Systematic Reviews and Meta-analyses (PRISMA) guidelines [17].

### Data sources and search strategy

We searched PubMed, Cochrane Library, and EMBASE databases for English or French reports published up to October 1, 2020. All reports of RCTs exploring the effect of n-3 PUFAs (eicosapentaenoic acid [EPA], docosahexaenoic acid [DHA], docosapentaenoic acid [DPA], alpha linolenic acid [ALA]) and n-6 PUFAs (LA, GLA) on disease activity, systemic inflammation, or pain over time compared to controls were included. We used the following search terms in PubMed, Cochrane Library, and EMBASE databases: ("Fatty Acids, Omega-3" OR “omega 3” OR “n-3 Fatty Acids” OR “n-3 Polyunsaturated Fatty Acid” OR “n-3 PUFA” OR “omega 3 Fatty Acids” OR "Fatty Acids, Omega-6" OR “omega 6” OR “n-6 Fatty Acids” OR “n-6 Polyunsaturated Fatty Acid” OR “n-6 PUFA” OR “Omega 6 Fatty Acids” OR “Polyunsaturated Fatty Acids”) AND (“Spondylitis, Ankylosing” OR ankylosis OR Spondylarthritis OR “Arthritis, Rheumatoid” OR “rheumatoid arthritis” OR “Arthritis, Psoriatic” OR “Psoriatic Arthritis”). We searched the reference lists of articles. We also collected data from the electronic abstract databases of the annual scientific meetings of the European Alliance of Associations for Rheumatology and American College of Rheumatology from 2009 to 2020 by using the term “omega-3,” “omega-6,” or “PUFA.”

### Study selection

The articles identified were evaluated independently by two investigators (JS and SM). “Two consecutives selections where performed, first by title and abstract and then in full text.” Any doubts about article selection were resolved by consensus after discussion with other investigators (CD and SM). The studied population comprised adults with rheumatic diseases (RA, ankylosing spondylitis [AS], psoriatic arthritis [PsA]); the intervention analyzed was oral supplementation of n-3 and/or n-6 PUFAs; all comparators were allowed in the control group (placebo, active comparator, no comparator); the outcomes retained were parameters of disease activity (duration of MS, visual analog scale [VAS] score for pain and activity; DAS28; TJC, SJC, HAQ score, erythrocyte sedimentation rate [ESR] and C-reactive protein [CRP] level). Only RCTs were included. We excluded citations with an unavailable full-text article and for meta-analysis, those with data not suitable for statistical analysis (no standard deviation or interquartile range for means or medians or intervention not limited to oral PUFA supplementation).

### Data extraction

Two investigators (SM and JS) independently examined participant (patients and control) characteristics and extracted all data by using a standardized data abstraction form. For all extracted data, the number of participants or a central value (mean or median) and variability (standard deviation or interquartile range) were obtained.

We extracted the number of patients who used PUFAs and the number of controls. For these populations, we extracted the type of rheumatic disease (RA, AS, or PsA), the mean change in MS duration, VAS pain and activity, DAS28, TJC, SJC, HAQ score, ESR, and CRP level after the intervention.

We also collected data on age and percentage of women. Interventions were classified by the source of PUFA (animal or vegetable), type of n-3 PUFAs (EPA, DHA, DPA, ALA) and n-6 PUFAs (LA, GLA), and dose and duration of supplementation. Any marine oil (such as seal oil, microalgae oil) was classified as “fish oil.”

### Quality assessment of the included studies

Risk of bias assessments were performed during data collection independently by two authors (SM and JS) and involved the Cochrane Collaboration’s Risk of Bias tool and the Jadad scale for each study. Records limited to abstracts were not assessed because of the paucity of available information. We classified the studies according to the Jadad score: ≥4 or <4.

### Statistical analyses

Baseline characteristics are summarized for each study sample and reported as the mean (standard deviation) or number (percentage) for continuous and categorical variables, respectively. The differences between the effect of n-3/n-6 PUFA supplementation and control on variables of disease activity over time were calculated as the standardized mean difference (SMD) by using the inverse-variance method. We chose the SMD because included studies could assess the same outcome but measure it in different ways. For example, TJC corresponded to the number of painful joints in recent studies and to Richie’s index in older studies. The SMD was interpreted according to the Cohen’s *d* as trivial, <0.2; small, 0.2–0.3; moderate, 0.5–0.8; and large, >0.8 [18].

Statistical heterogeneity was assessed by examining forest plots, SMD, and confidence intervals (CIs) and calculating the *I*^2^ index, the most common metric for measuring the magnitude of between-study heterogeneity [19]. *I*^2^ values range from 0 to 100% and are considered low at <25%, modest at 25–50%, and high at >50%. Random-effects models assuming between- and within-study variability (DerSimonian and Laird approach) were used if heterogeneity was present; otherwise, a fixed-effects model was used. When possible (sufficient sample size), meta-regression analysis was used to study the relation between effect sizes of n-3/n-6 PUFA consumption, SMDs of TJC and SJC, VAS pain, HAQ score, DAS28, CRP level and ESR, MS duration, and study characteristics (Jadad score ≥4 or <4, type, source and dose of PUFA, and type of rheumatic disease). Subgroup analyses were also performed according to these characteristics.

Finally, to check the robustness of the results, we performed sensitivity analyses excluding studies not evenly distributed around the base of the funnel plots and according to Egger’s test (publication bias). Two-sided *p*<0.05 was considered statistically significant. All statistical analyses involved using Stata 15 (StataCorp, College Station, TX, USA). The PRISMA checklist is in the appendix (Additional file 1). The protocol was registered at PROSPERO (CRD42021253685).

## Results

**Fig. 1:**
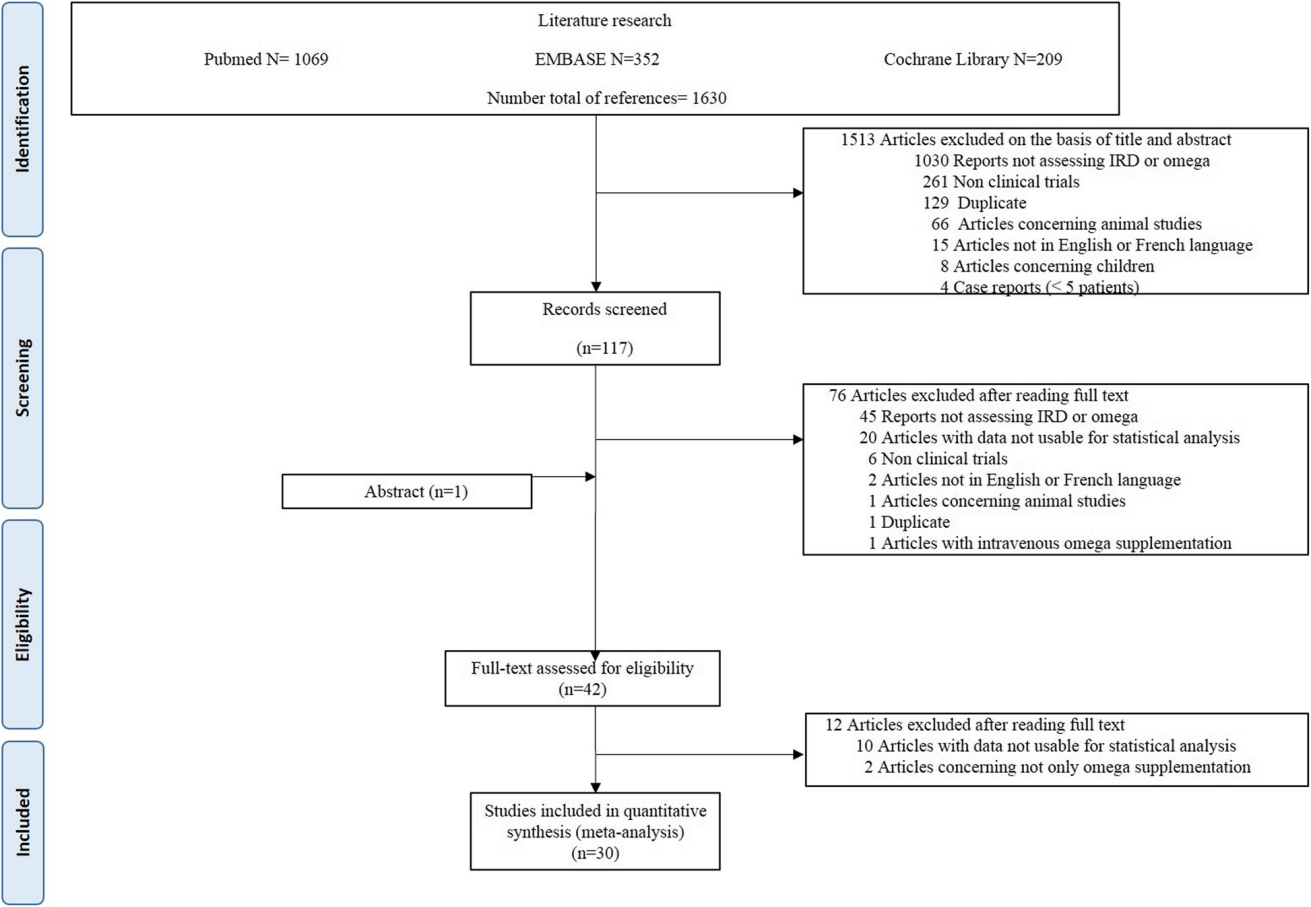
Flow diagram of the literature search process

A total of 1630 citations were obtained from the initial search and screened for titles and abstracts (Fig. 1).

After excluding articles not meeting the inclusion criteria, 117 full papers were screened and 42 were included in the systematic review. Among them, 30 had sufficient data and were included in the meta-analysis. Characteristics of the included studies are presented in Table 1 (and Additional file 2).

**Table 1:** Characteristics of the publications included in the meta-analysis by source and type of polyunsaturated fatty acid (PUFA)

Study	Disease	No. of participants		PUFA source	PUFA dose (g/day)							Control	Duration (weeks)	Jadad score	Measures
		PUFA	Total		EPA	DHA	DPA	ALA	Total n-3 intake	GLA	Total n-6 intake				
Araujo 2014 [20]	RA	11	37	Fish oil								Not mentioned	24	Abstract	TJ, SJ, activity, DAS28, CRP, ESR
Berbert 2005 [21]	RA	30	43	Fish oil					3			Soy oil	24	2	MS, pain, activity, CRP, ESR
Brzeski 1991 [22]	RA	19	40	Vegetable oil						0.5	0.5	Olive oil capsules	24	3	TJ, MS, pain
Cleland 1988 [23]	RA	23	23	Fish oil					5.2			Olive oil capsules	12	4	TJ, SJ, MS, pain
Das Gupta 2009 [24]	RA	50	100	Fish oil					3			No special diet or intervention	12	1	TJ, SJ, pain, DAS28, CRP, ESR
Dawczynski 2009 [25]	RA	14	26	Fish oil	0.7	0.4	0.1	1.1	2.3			Dairy with comparable fat content	24	3	TJ, SJ, DAS28, CRP, ESR
Dawczynski 2011 [26]	PsA+RA	14	47	Fish oil					3.0			Olive oil capsules	12	4	DAS28
Dawczynski 2018 [27]	RA	25	25	Fish oil		2.1			2.1			Sunflower oil capsules	10	3	TJ, SJ, DAS28, CRP, ESR
Galarraga 2008 [28]	RA	49	97	Fish oil	1.5	0.7			2.2			Air-filled capsules	36	4	Pain, DAS28, CRP, HAQ
Geusens 1994 [29]	RA	21	60	Fish oil					1.3			Olive oil capsules	48	3	TJ, pain, activity
		19							2.6						
Khaleghi 2005 [30]	RA	10	39	Fish oil					10			Corn oil	8	Abstract	TJ, SJ
Kolahi 2010 [31]	RA	40	83	Fish oil	0.2	0.1			0.3			Capsules without n-3 PUFAs	24	3	DAS28, CRP
Kremer 1990 [32]	RA	20	49	Fish oil	1.9	1.3			3.2			Olive oil capsules	24	2	TJ, SJ, MS, pain, activity
		17			3.8	2.5			6.3						
Kremer 1995 [33]	RA	23	49	Fish oil					9.1			Corn oil capsules	30	2	TJ, SJ, MS, pain, activity
Kremer 1985 [34]	RA	23	44	Fish oil	1.7	1.1			2.8			Diet low in PUFA/SFA plus paraffin capsules	12	4	TJ, SJ, MS
Kristensen 2018 [35]	PsA	72	143	Fish oil	1.5	1.5			3.0			Olive oil capsules	24	5	TJ, SJ, pain, DAS28, HAQ
Leventhal 1993 [36]	RA	19	37	Vegetable oil						1.4	1.4	Cottonseed oil capsules	24	2	TJ, SJ, MS, pain, activity, ESR
Madland 2006 [37]	PsA	20	40	Fish oil	2.4	2.6	1.1		6.1			Soil oil capsules	0.5	2	TJ, SJ, pain, activity, ESR, HAQ
Magaro 1992 [38]	RA	10	20	Fish oil	1.5	1.0			2.5			No special diet or intervention	6	1	MS, pain, ESR
Nielsen 1992 [39]	RA	29	57	Fish oil	2.0	1.2			3.2			Capsules with fat composition as the average Danish diet	12	4	TJ, SJ, CRP, ESR
Nordström 1995 [40]	RA	11	22	Vegetable oil				9.6	9.6			Safflower oil	24	2	TJ, pain, activity, CRP, ESR
Park 2013 [41]	RA	41	81	Fish oil	2.1	1.2			3.3			Sunflower oil with oleic acid capsules	16	2	Pain, activity, CRP, HAQ
Skoldstam 1992 [42]	RA	22	46	Fish oil	1.7	1.1			2.8			Maize/olive/peppermint oil capsules	24	4	Pain, activity, CRP, ESR
Sundrarjun 2004 [43]	RA	23	60	Fish oil					3.4			Diet low in PUFA plus placebo capsules	24	3	TJ, SJ, pain, activity, CRP, ESR, HAQ
Sundström 2006 [44]	AS	9	18	Fish oil					1.9			Low dose vs high dose	21	1	ESR
Tulleken 1990 [45]	RA	12	24	Fish oil	2.0		1.3		3.3			Coconut oil capsules	24	4	TJ, SJ, pain, CRP, ESR
Veale 1994 [46]	PsA	19	38	Mix oil	0.2	0.1			0.3	0.5	0.5	Liquid paraffin and vitamin E capsules	36	2	TJ
Veselinovic 2017 [47]	RA	20	60	Fish oil	1.0	1.5			2.5			No special diet or intervention	12	2	TJ, SJ, pain, DAS28, CRP, ESR
Volker 2000 [48]	RA	13	26	Fish oil					1.7			Diet low in u-6 PUFAs plus corn and olive oil	15	3	TJ, SJ, pain, activity, CRP, ESR, HAQ
Zurier 1996 [49]	RA	28	56	Vegetable oil						2.8	2.8	Sunflower seed oil	24	4	TJ, SJ, MS, pain, activity, HAQ

*EPA* eicosapentaenoic acid (C20:5n3), *DHA* docosahexaenoic acid (C22:6n3), *DPA* docosapentaenoic acid (C22:5n3), *ALA* linolenic acid (C18:3n3), *GLA* gamma-linolenic acid (C18:3n6), *n-3* omega-3, *n-6* omega-6, *PUFA* polyunsaturated fatty acid, *EPO* evening primrose oil, *RA* rheumatoid arthritis, *PsA* psoriatic arthritis, *AS* ankylosing spondylitis, *activity* patient activity on visual analog scale, *CRP* C-reactive protein, *DAS* Disease Activity Score, *ESR* erythrocyte sedimentation rate, *HAQ* Health Assessment Questionnaire, *MS* morning stiffness, *NSAID* non-steroidal anti-inflammatory drug, *SJ* swollen joint count, *TJ* tender joint count

The assessment of the quality of the 40 included studies (2 abstracts [20] and [30] were excluded from the quality analysis) revealed a selective reporting bias. The primary outcome was rarely defined in these studies (risk of bias according to the Cochrane Collaboration Risk of Bias tool summarized in Additional file 3). The median Jadad score for these 40 studies was 3 (interquartile range 2–4). Twelve (30%) studies had a Jadad score ≥ 4.

We included 30 of the 42 studies in the meta-analysis (Table 1) (710 patients receiving PUFAs and 710 controls); 10 studies were excluded because data were lacking on standard deviation, mean, or number of patients included (Additional file 2). Despite an e-mail sent to authors, we did not obtain usable data for these studies. Two other studies were excluded because of mixed supplementation of n-3/n-6 PUFAs and zinc or other nutrients (Additional file 2).

At baseline, the mean age of the 710 patients starting PUFA supplementation was 53.3 years, 73.5% were women, and disease activity was high (weighted mean±sd TJC: 12.2 ± 6.3, SJC: 7.8 ± 3.3, DAS28: 4.3 ± 0.7, CRP level: 16.4 ± 11.1 mg/l). The characteristics of patients included in the meta-analysis are presented in Additional file 4. Disease duration and baseline disease activity were globally similar between the intervention and control groups.

### Overall effects of PUFA supplementation on disease activity

When pooling all studies and timepoints, all clinical parameters of disease activity were significantly improved by oral PUFA supplementation. Improvement was high for VAS activity; moderate for VAS pain, DAS28, and HAQ score; and low for MS duration, SJC, and TJC (Table 2).

**Table 2:** Effect of oral PUFA supplementation on parameters of inflammatory rheumatic disease activity compared to control

Parameters	Effect of PUFA supplementation over time				I^**2**^
	After 1 month of treatment	After 3 months of treatment	After 6 months of treatment	Overall effect^**a**^	
Tender joints	*N*=3 *n*=122 (62+60) − 0.34 [−0.75, 0.08]	*N*=15 *n*=606 (305+301) − 0.26 [−0.57, 0.05]	*N*=9 *n*=408 (206+202) − 0.39 [−0.78, −0.000]	− 0.31 [−0.52, −0.10] *p*=0.004	67%R
Swollen joints	*N*=3 *n*=122 (62+60) − 0.09 [−0.70, 0.51]	*N*=11 *n*=467 (237+230) − 0.32 [−0.55, −0.09]	*N*=7 *n*=330 (168+162) − 0.11 [−0.42, 0.19]	− 0.22 [−0.39, −0.05] *p*=0.01	39%R
Morning stiffness duration	*N*=2 *n*=56 (29+27) − 0.47 [−1.60, 0.66]	*N*=11 *n*=509 (258+251) − 0.19 [−0.36, −0.01]	*N*=6 *n*=192 (100+92) − 0.77 [−1.39, −0.16]	− 0.38 [−0.61, −0.15] *p*=0.001	56%R
VAS pain	*N*=3 *n*=106 (53+53) − 1.46 [−4.33, 1.41]	*N*=13 *n*=597 (300+297) − 0.57 [−0.99, −0.15]	*N*=11 *n*=549 (279+270) − 0.32 [−1.24, 0.60]	−0.48 [−0.95, −0.01] *p*=0.04	93%R
VAS activity	*N*=2 *n*=86 (43+43) − 1.04 [−2.47, 0.38]	*N*=7 *n*=266 (136+130) − 1.08 [−2.16, −0.009]	*N*=8 *n*=280 (143+137) − 1.04 [−1.92, −0.16]	− 1.05 [−1.63, −0.47] *p*<0.001	91%R
DAS28		*N*=7 *n*=419 (209+210) − 0.41 [−0.78, −0.04]	*N*=3 *n*=255 (126+129) − 0.22 [−0.47, 0.02]	−0.36 [−0.60, −0.11] *p*=0.005	58% R
CRP	*N*=1 *n*=46 (23+23) −0.52 [−1.10, 0.07]	*N*=13 *n*=669 (335+334) 0.15 [−0.44, 0.74]	*N*=5 *n*=238 (118+120) −0.62 [−1.17, −0.06]	−0.08 [−0.53, 0.36] *p*=0.71	90%R
ESR	*N*=3 *n*=106 (53+53) − 0.47 [−0.86, −0.08]	*N*=11 *n*=426 (214+212) − 0.25 [−0.50, 0.002]	*N*=6 *n*=186 (92+94) −0.23 [−0.52, 0.06]	− 0.28 [−0.43, −0.13] *p*<0.001	44% R
HAQ	*N*=2 *n*=86 (43+43) −0.15 [−0.93, 0.63]	*N*=3 *n*=204 (103+101) −1.48 [−2.92, −0.04]	*N*=4 *n*=316 (160+156) −0.48 [−0.87, −0.09]	− 0.66 [−1.11, −0.21] *p*=0.004	85%R

Data are standardized mean difference [95% confidence interval]

*VAS* visual analog scale, *DAS28* Disease Activity Score in 28 joints, *ESR* erythrocyte sedimentation rate, *CRP* C-reactive protein, *HAQ* Health Assessment Questionnaire, *N* number of studies, *n* number of patients, *n=Z (X+Y) Z*=number of participants (*X*=number of patients receiving PUFAs + *Y*=number of controls), *R* random effects analysis, *F* fixed effects analysis

^a^ When pooling all studies and timepoints

We found a conflicting effect of oral PUFA supplementation on factors of systemic inflammation, with a significant decrease in ESR but not CRP level (Table 2). Similar results were found when including only RA patients, who represented most of the studied population (26/30 studies: 1192/1420 patients) (Additional file 5, Fig. 2, Additional files 6, 7, 8, 9, 10, 11, 12, 13, and 14).

**Fig. 2:**
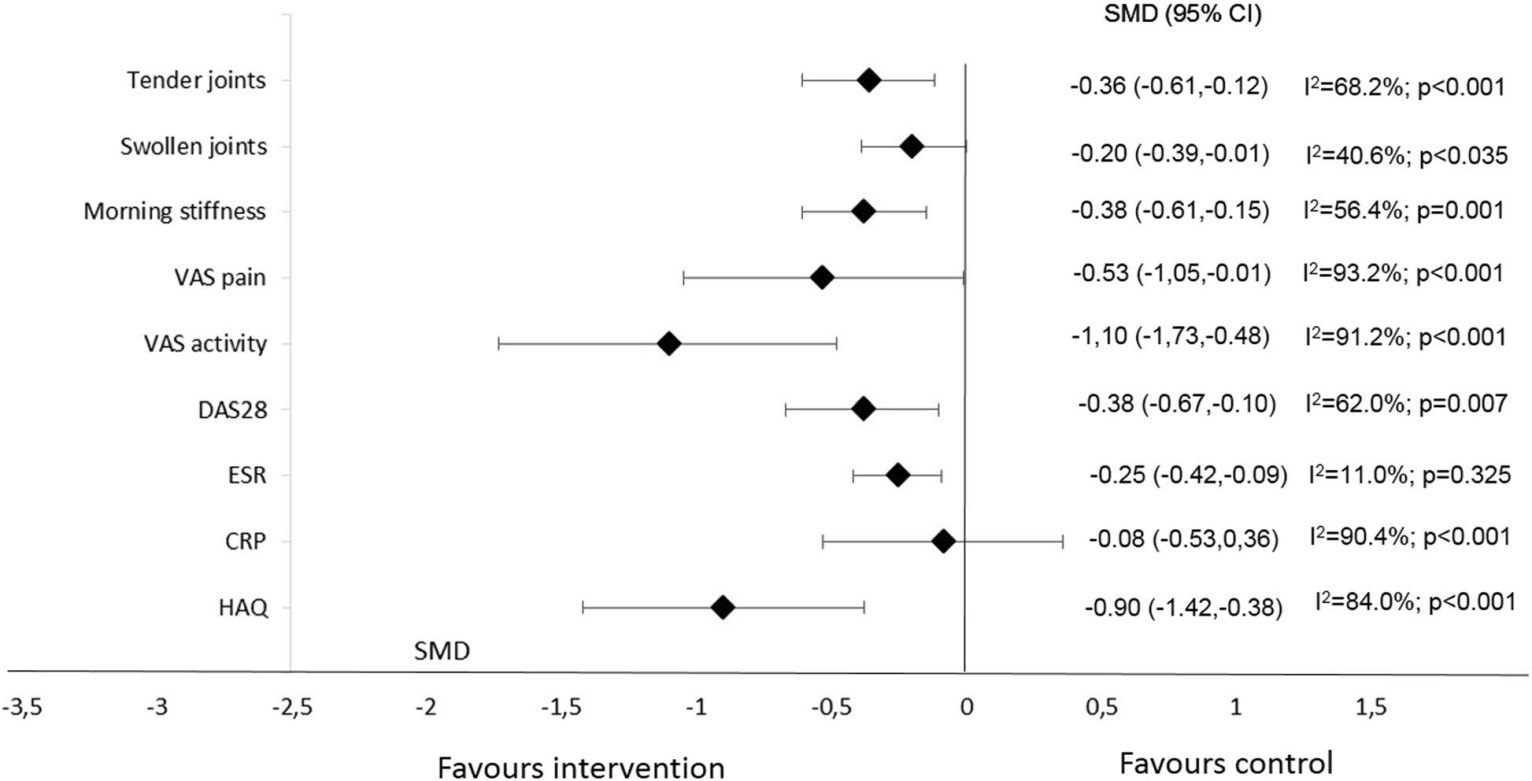
Overall effect of PUFA supplementation in rheumatoid arthritis (RA) patients compared to controls (pooling of all timepoints). VAS visual analog scale, DAS28 Disease Activity Score in 28 joints, ESR erythrocyte sedimentation rate, CRP C-reactive protein, HAQ Health Assessment Questionnaire

Of note, the analysis of treatment effects according to disease activity in RA patients showed similar results. Indeed, taking into account only studies with a mean DAS28 > 3.2, the same activity parameters were improved by supplementation, except for the MS duration which was no longer significant (Additional file 15).

**Fig. 3:**
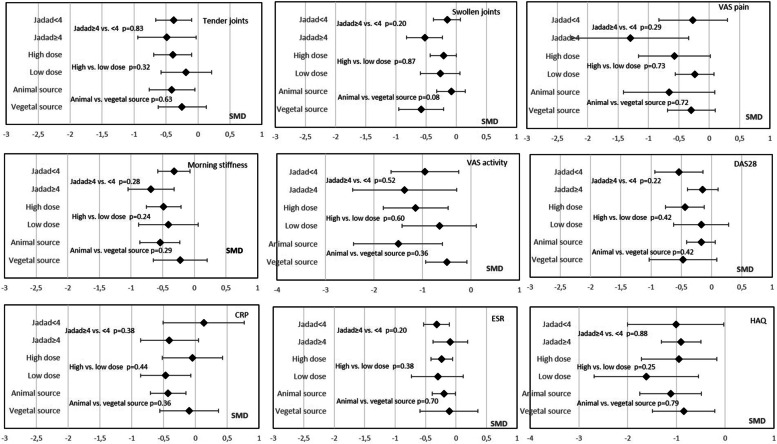
Meta-regression analysis of RA patients with oral PUFA supplementation. Data are standardized mean difference (SMD) (95% CI). VAS visual analog scale, DAS28 Disease Activity Score in 28 joints, ESR erythrocyte sedimentation rate, CRP C-reactive protein, HAQ Health Assessment Questionnaire

Sensitivity analyses according to funnel plots and metabias revealed no difference in MS duration or VAS activity after excluding Kremer et al. [34] and Geusens et al. [29] (Additional file 16). Likewise, Egger’s test was statistically significant for CRP analysis (*p*=0.047) because of the results of the Das Gupta et al. study [24]. Sensitivity analysis excluding this study revealed a significant improvement in CRP level (overall pooled SMD for CRP = −0.37 [95% CI −0.60; −0.13]; *p*=0.002). Furthermore, meta-regression revealed no significant effect of overall results for the Jadad score (Fig. 3).

Of the studies included in the systematic review but not in the meta-analysis, only 4 out of 10 and 2 out of 4 showed a significant positive effect (or a trend) for TJC and SJC, respectively. All positive studies included high doses of n-3 PUFAs (2 g per day) and/or mussels, as did half of the negative studies. Surprisingly, only one out of 7 studies (mussel-based diet) showed an effect on pain and 3 out of 8 a positive effect on MS; none found significant results regarding ESR (among 6 studies), CRP level (5 studies), and HAQ score (3 studies). Two out of 4 studies showed a significant decrease in DAS28; both included mussels as an n-3 PUFA source (data not shown).

### Effect of oral PUFA supplementation on disease activity over time

None of the clinical and biological parameters was significantly changed after 1 month of supplementation (1 to 3 studies, including 46 to 122 patients), whereas all clinical parameters but TJC were significantly improved after 3 months (3 to 15 studies, including 204 to 669 patients) (Table 2). Similarly, when focusing on RA patients, 1-month supplementation significantly decreased only VAS activity (1 to 2 studies, including 46 to 82 patients), whereas 3-month supplementation significantly decreased all clinical parameters and no biological parameters (ESR, CRP level) (3 to 14 studies, including 204 to 669 patients) (Additional file 5, Fig. 2).

### Effect of oral PUFA supplementation on RA disease activity by PUFA type (n-3 or n-6)

**Fig. 4:**
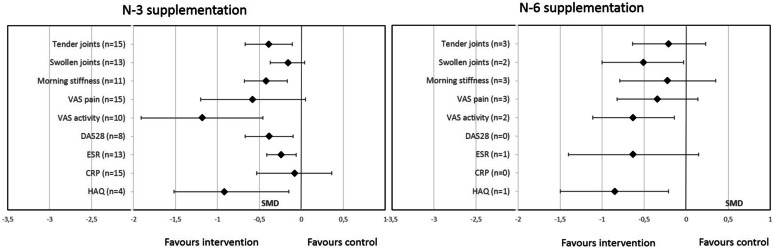
Effect of oral PUFA supplementation on RA disease activity by PUFA type (n-3 or n-6). Data are standardized mean difference (SMD) (95% CI). VAS visual analog scale, DAS28 Disease Activity Score in 28 joints, ESR erythrocyte sedimentation rate, CRP C-reactive protein, HAQ Health Assessment Questionnaire

Because RA patients represented much of the studied population, to increase homogeneity, we focused a subgroup analysis on studies including RA patients. All but 3 studies used n-3 PUFA supplementation. Analyses focusing on n-3 PUFA supplementation showed a significant improvement in all parameters, except SJC, VAS pain, and CRP level (Fig. 4). Supplementation with n-6 PUFAs significantly decreased SJC, VAS activity, and HAQ score (Fig. 4).

### Effect of oral PUFA supplementation on RA disease activity by PUFA source (vegetable or animal)

**Fig. 5:**
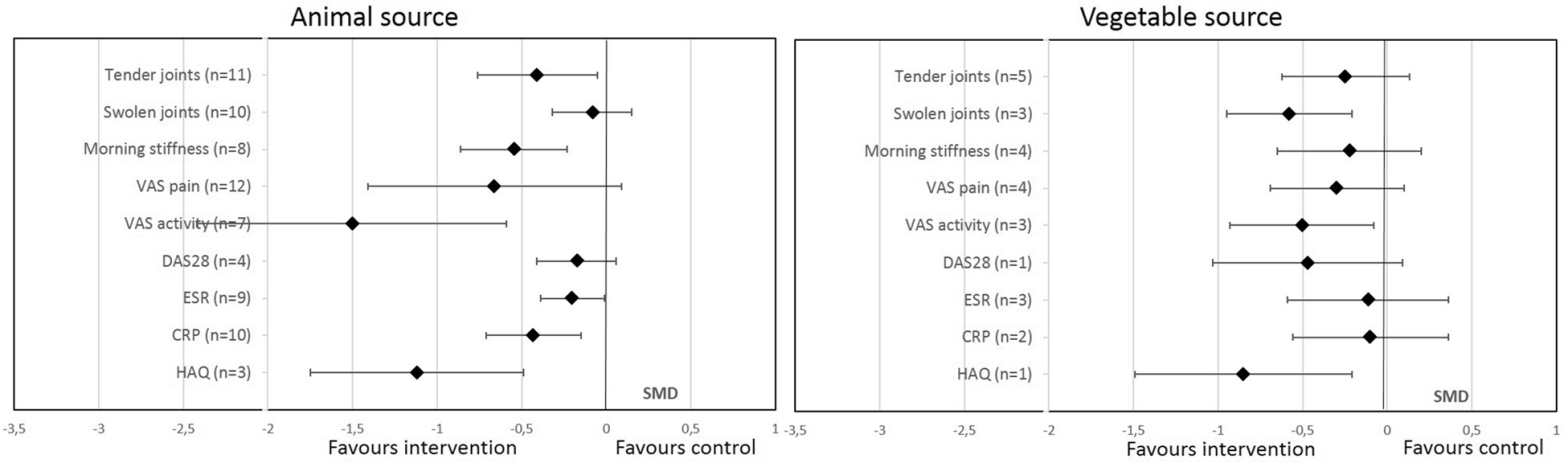
Effect of oral PUFA supplementation on RA disease activity by source (vegetable or animal). Data are standardized mean difference (SMD) (95% CI). VAS visual analog scale, DAS28 Disease Activity Score in 28 joints, ESR erythrocyte sedimentation rate, CRP C-reactive protein, HAQ Health Assessment Questionnaire

Subgroup analysis revealed that animal-derived PUFA supplementation significantly decreased TJC, VAS activity, MS duration, HAQ score, CRP level, and ESR but not SJC, DAS28, or VAS pain (Fig. 5). Conversely, vegetable-derived PUFA supplementation significantly decreased only SJC, VAS activity, and HAQ score (Fig. 5).

However, a meta-regression comparing the 2 sources did not show any significant difference (Fig. 3).

### Effect of oral PUFA supplementation on RA disease activity by dosage

**Fig. 6:**
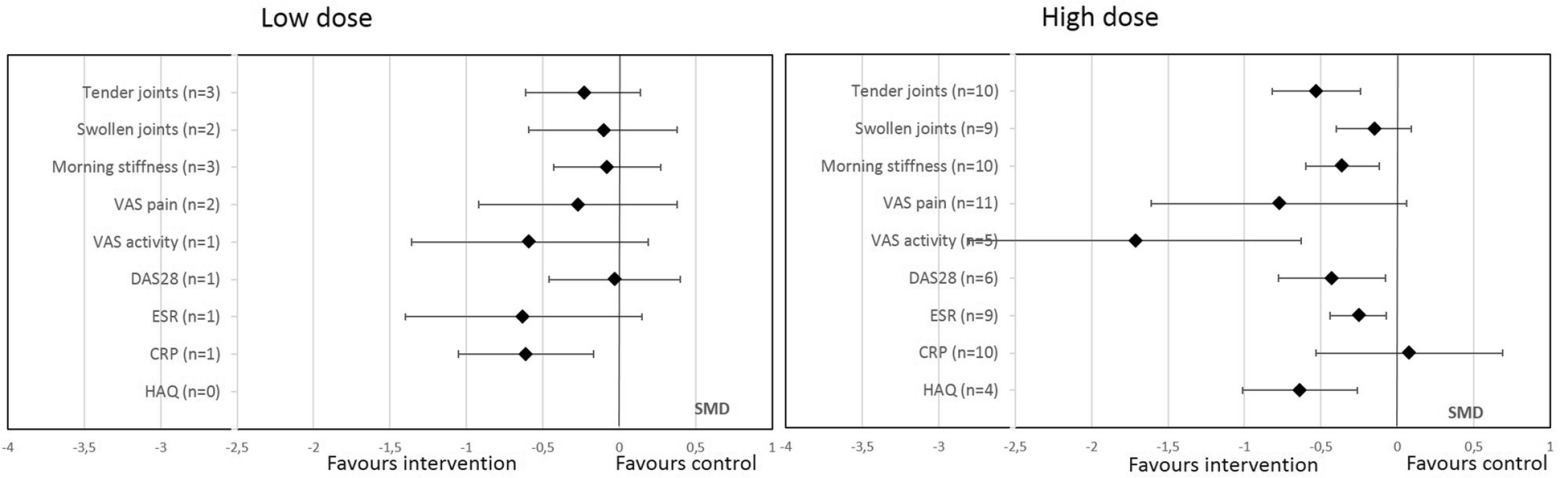
Effect of oral PUFA supplementation on RA disease activity by dosage (> or < 2 g/day). Data are standardized mean difference (SMD) (95% CI). VAS visual analog scale, DAS28 Disease Activity Score in 28 joints, ESR erythrocyte sedimentation rate, CRP C-reactive protein, HAQ Health Assessment Questionnaire

Subgroup analysis revealed that high-dose PUFA supplementation (≥ 2 g/day) significantly decreased TJC, VAS activity, MS duration, DA28, HAQ score, and ESR but not SJC, VAS pain, or and CRP level (Fig. 6). Low-dose PUFA supplementation (< 2 g/day) significantly decreased only CRP level (Fig. 6).

Meta-regression comparing the 2 doses did not show any significant difference (Fig. 3).

### Tolerance

Of the 30 studies in the meta-analysis, only 15 mentioned the collection of adverse events. Of these, no serious adverse events were reported, 4 reported no adverse events, and 11 reported moderate gastrointestinal disturbances (diarrhea, constipation, flatulence). No difference between the two groups (intervention vs. control) was found.

## Discussion

The management of IRD is mostly based on a drug-based approach mainly targeting a reduction in inflammation to prevent joint damage, morbidity, and mortality. However, patients perceive reduction of pain and fatigue and maintenance of physical function as major unmet needs [50–52]. Thus, despite increasingly effective management, patients express a significant number of unmet needs, with the need for self-management of the disease, particularly through diet. This meta-analysis included 30 reports of RCTs assessing the effect of oral PUFA supplementation (n-3 and/or n-6, fish and/or vegetable oil) on IRD activity. Oral PUFA supplementation had a beneficial effect on most criteria for disease activity across all IRDs (RA, PsA, and AS) even though most studies involved RA patients. Improvement was large for VAS activity; moderate for VAS pain, DAS28, and HAQ level; and small for MS duration, SJC, and TJC. These beneficial effects seemed to be observed only for a minimum of 3 months of supplementation because none of the clinical and biological parameters was significantly changed after 1 month of supplementation, whereas all clinical parameters (but TJC) were significantly improved after 3 months and 6 months. No clinical trials explored supplementation duration above 6 months in established RA but there is evidence that long-term supplementation may be well tolerated [2].

We found a significant decrease in only ESR but not CRP level. Hence, DAS28 improvement seemed linked more to a significant decrease in SJC and VAS activity than a significant improvement in biological systemic inflammation. Finally, these improvements are not associated with significant side effects. Only moderate gastrointestinal (GI) disorders have been reported, which may be related to the capsule composition. These GI disturbances may nevertheless be a cause of poor compliance.

### Subgroup analyses

The novelty of this meta-analysis is the subgroup analyses by duration, type, origin, and dose of PUFAs. Indeed, for the Lee et al. meta-analysis published in 2012 [13], including studies of only ≥ 2.3-g/day supplementation (10 RCTs), results were contradictory with previous and subsequent meta-analyses. The Lee et al. meta-analysis found a beneficial effect of supplementation on only NSAID use and no effect on TJC, SJC, and MS duration, unlike the meta-analyses of Gioxari et al., Goldberg et al., and Fortin et al. [11, 12, 15]. These results favored a possible dose-effect of PUFAs with a homeopathic threshold at 2.3 g/day. Our meta-analysis, which included many more RCTs, found effects on these different parameters that seemed to be independent of PUFA dose. Nevertheless, most studies (24 of 29 with available PUFA dose) assessed a dose >2 g/day. Thus, the strongest evidence of PUFA efficacy remains with supplementation > 2 g/day. It is noteworthy that higher dose supplementation has been associated with an increased risk of bleeding and therefore it is recommended not to exceed this dosage [53, 54]. On subgroup analysis by origin of PUFA (animal or vegetable source), more parameters were significantly improved with fish oil than a vegetable source (6/9 vs 3/9). However, most studies concerned fish-oil supplementation (23/31) and the difference might also be explained by a lack of power for the vegetable subgroup analysis.

The most recent meta-analysis was published in 2018 [15]. It included 20 reports of RCTs that focused exclusively on n-3 PUFA supplementation in RA. The main results showed a significant improvement in TJC, MS duration, pain, HAQ score, and ESR, with no effect on CRP level, SJC, and DAS28. Therefore, our results are broadly consistent (except for DAS28 and SJC) and strengthen these results with the analysis of 11 additional RCTs.

### Clinical implications

These positive results, combined with minor side effects, support the need for nutritional support in addition to standard drug therapy in IRD patients. Beyond the impact on disease activity, the beneficial effects could be cardiovascular as well as for high cardiovascular mortality and morbidity. A recent meta-analysis reported beneficial effects of PUFA supplementation on cardiovascular disease with at least a dose of 2 g/day [55]. However, as compared with the large evidence available for low doses, the evidence for higher doses (2–4 g/day) is weak and the beneficial effects of n-3 PUFAs on cardiovascular risk remain debated [56]. The STRENGTH trial of statin-treated participants with high cardiovascular risk, hypertriglyceridemia and low level of high-density lipoprotein cholesterol found no beneficial effect of 4 g/day of a carboxylic acid formulation of n-3 PUFAs (a combination of EPA and DHA) as compared with corn oil [56]. However, in the same high-risk, statin-treated population, the REDUCE-IT trial found a 25% reduction in a composite endpoint of cardiovascular events in the n-3–treated group (icosapent ethyl) versus the mineral oil-treated group [54]. Hence, the effect of PUFA supplementation on cardiovascular events in IRD patients remains to be explored.

### Limitations

Our study has limitations. First, the number of patients with inflammatory rheumatic disease in our meta-analysis was quite low. Most studies included fewer than 30 patients receiving PUFAs, which could be considered not sufficient to draw strong conclusions for the effect on disease activity. However, the inclusion of studies represented more than 700 patients and 700 controls, which is appropriate for a meta-analysis. Moreover, we included only RCTs and performed a subgroup analysis by Jadad score for quality, which revealed no difference in results. Another limitation is related to the publication bias, positive studies being more likely to be published than negative ones. However, we searched for relevant abstracts in European and American congresses and found no other references. In addition, because most studies in the meta-analysis focused on patients with RA, we cannot extrapolate these results to AS and PsA patients. Studies of these specific diseases are needed to increase the level of evidence.

The main limitation of this meta-analysis is the inclusion of very heterogeneous studies regarding the control population (placebo, active comparator, no comparator) and supplementation (dose, type). This limitation explains the substantial heterogeneity observed in the analysis.

## Conclusions

The findings of this systematic review and meta-analysis suggest that PUFA supplementation, especially omega-3 from animal source >2 g/day, seems of interest in terms of disease activity for IRD, with good tolerance, in addition to disease-modifying antirheumatic drug prescription.

## Supplementary Information

**Additional file 1.** Prisma Checklist.

**Additional file 2.** Characteristics of studies not included in the meta-analysis by source of PUFA.

**Additional file 3.** Risk of bias according to the Cochrane Collaboration Risk of Bias tool.

**Additional file 4.** Population baseline characteristics of studies included in the meta-analysis.

**Additional file 5.** Effect of PUFA supplementation compared to control on parameters of rheumatoid arthritis.

**Additional file 6.** Forest plot of effect of oral PUFA supplementation on tender joint count in RA.

**Additional file 7.** Forest plot of effect of oral PUFA supplementation on swollen joint count in RA.

**Additional file 8.** Forest plot of effect of oral PUFA supplementation on morning stiffness duration in RA.

**Additional file 9.** Forest plot of effect of oral PUFA supplementation on patient-reported pain (VAS) in RA.

**Additional file 10.** Forest plot of effect of oral PUFA supplementation on patient-reported disease activity (VAS) in RA.

**Additional file 11.** Forest plot of effect of oral PUFA supplementation on DAS28 in RA.

**Additional file 12.** Forest plot of effect of oral PUFA supplementation on ESR in RA.

**Additional file 13.** Forest plot of effect of oral PUFA supplementation on CRP level in RA.

**Additional file 14.** Forest plot of effect of oral PUFA supplementation on HAQ score in RA.

**Additional file 15.** Effect of oral PUFA supplementation on RA disease activity by disease activity (DAS28< or > 3.2).

**Additional file 16.** Funnel plots of publication bias.

## Data Availability

The datasets used and analyzed during the current study are available from the corresponding author on reasonable request.

## Abbreviations

ALA: Alpha linolenic acid
AS: Ankylosing spondylitis
CRP: C-reactive protein
DAS28: Disease Activity Score in 28 joints
DHA: Docosahexaenoic acid
DPA: Docosapentaenoic acid
EPA: Eicosapentaenoic acid
ESR: Erythrocyte sedimentation rate
GLA: Gamma-linolenic acid
HAQ: Health Assessment Questionnaire
IRD: Inflammatory rheumatic diseases
LA: Linoleic acid
MS: Morning stiffness
n-3/n-6: Omega 3/6
NSAID: Non-steroidal anti-inflammatory drug
PUFA: Polyunsaturated fatty acids
PsA: Psoriatic arthritis
RA: Rheumatoid arthritis
RCT: Randomized controlled trial
SJC: Swollen joint count
TJS: Tender joint count
VAS: Visual analog scale
