# Supplementary material for: Impact of type and dose of oral polyunsaturated fatty acid supplementation on disease activity in inflammatory rheumatic diseases: a systematic literature review and meta-analysis
Source: Arthritis Res Ther. 2022 May 7;24:100. doi: 10.1186/s13075-022-02781-2 (PMC9077862; doi:10.1186/s13075-022-02781-2)
Supplement: Supplementary file 2 — Additional file 2. Characteristics of studies not included in the meta-analysis by source of PUFA. [file 13075_2022_2781_MOESM2_ESM.docx]

**Additional file 2. Characteristics of studies not included in the meta-analysis by source of PUFA**

| Study | Disease | No. of participants | | PUFA source | PUFA dose (g/day) | | | | | | | | Control | Duration (weeks) | Jadad score | Measures |
| --- | --- | --- | --- | --- | --- | --- | --- | --- | --- | --- | --- | --- | --- | --- | --- | --- |
|  |  | PUFA | Total |  | EPA | DHA | DPA | ALA | Total n-3 intake | GLA | LA | Total n-6 intake |  |  |  |  |
| Belch 1988 (57) | RA | 16 | 49 | EPO |  |  |  |  |  | 0.5 |  | 0.5 | Liquid paraffin | 52 | 4 | MS, pain, NSAID consumption, CRP, ESR |
| Espersen 1992 (58) | RA | 18 | 32 | Fish oil | 2.0 | 1.2 |  |  | 3.2 |  |  |  | Capsules with fat composition as the average Danish diet | 12 | 2 | Ritchie’s articular index |
| Fu 2015 (59) | RA | 18 | 42 | Hard-shelled mussels |  |  |  |  |  |  |  |  | Corn-oil capsules | 24 | 5 | TJ, pain, ESR |
| Huskisson 1981 (60) | RA | 30 | 30 | Seatone |  |  |  |  |  |  |  |  | Air-filled capsules | 4 | 1 | TJ, SJ, pain, HAQ, DAS28, EULAR response, CRP, ESR |
| Larkin 1985 (61) | RA | 20 | 35 | Green-lipped mussel |  |  |  |  |  |  |  |  | Air-filled capsules | 24 | 3 | Ritchie’s articular index, pain, ESR |
| Lau 1993 (62) | RA | 32 | 64 | Fish oil | 1.7 | 1.1 |  |  | 2.8 |  |  |  | Air-filled capsules | 52 | 2 | TJ, MS, pain, SJ, pain, NSAID consumption, CRP, ESR |
| Lindqvist 2018 (63) | RA | 53 | 53 | Mussels |  |  |  |  |  |  |  |  | Vegetarian meal | 11 | 3 | TJ, SJ, pain, MS, DAS28, CRP, ESR |
| Magaro 1988 (64) | RA | 6 | 12 | Fish oil | 1.5 | 1.0 |  |  | 2.5 |  |  |  | Diet high in saturated fatty acids | 4 | 1 | MS, Ritchie’s articular index, pain, ESR |
| Proudman 2015 (65) | RA | 86 | 139 | Fish oil |  |  |  |  | 5.5 |  |  |  | Low dose vs high dose | 52 | 5 | NSAID consumption, HAQ, activity |
| Rajaei 2015 (66) | RA | 30 | 60 | Fish oil | 1.8 | 2.1 |  |  | 3.9 |  |  |  | Starch capsules | 12 | 2 | TJ, SJ, pain, NSAID consumption, CRP, ESR |
| Remans 2004 (67) | RA | 33 | 66 | Mix oil | 1.4 | 0.2 | 0.04 | 0.02 | 1.6 |  |  |  | Liquid vehicle | 16 | 3 | TJ, SJ, pain, DAS28, CRP, ESR |
| Van der Tempel 1990 (68) | RA | 16 | 16 | Fish oil | 2.0 | 1.3 |  |  | 3.3 |  |  |  | Coconut oil | 12 | 2 | Ritchie’s articular index |

EPA= eicosapentaenoic acid (C20:5n3); DHA= docosahexaenoic acid (C22:6n3); DPA= docosapentaenoic acid (C22:5n3); ALA= alpha linolenic acid (C18:3n3); GLA= gamma-linolenic acid (C20:3n6); LA= linoleic acid (C18:2n6); n-3= omega-3; n-6= omega-6; PUFA= polyunsaturated fatty acid; EPO= evening primrose oil; RA= rheumatoid arthritis; PsA= psoriatic arthritis; AS= ankylosing spondylitis; activity= patient activity on visual analog scale; CRP= C-reactive protein; DAS: disease activity score; ESR= erythrocyte sedimentation rate; HAQ= Health Assessment Questionnaire; MS= morning stiffness; NSAID= non-steroidal anti-inflammatory drugs; SJ= swollen joint count; TJ= tender joint count
