# Supplementary figures and images for: Impact of type and dose of oral polyunsaturated fatty acid supplementation on disease activity in inflammatory rheumatic diseases: a systematic literature review and meta-analysis
Source: Arthritis Res Ther. 2022 May 7;24:100. doi: 10.1186/s13075-022-02781-2 (PMC9077862; doi:10.1186/s13075-022-02781-2)

**Additional file 3 Risk of bias according to the Cochrane Collaboration Risk of Bias tool**

**
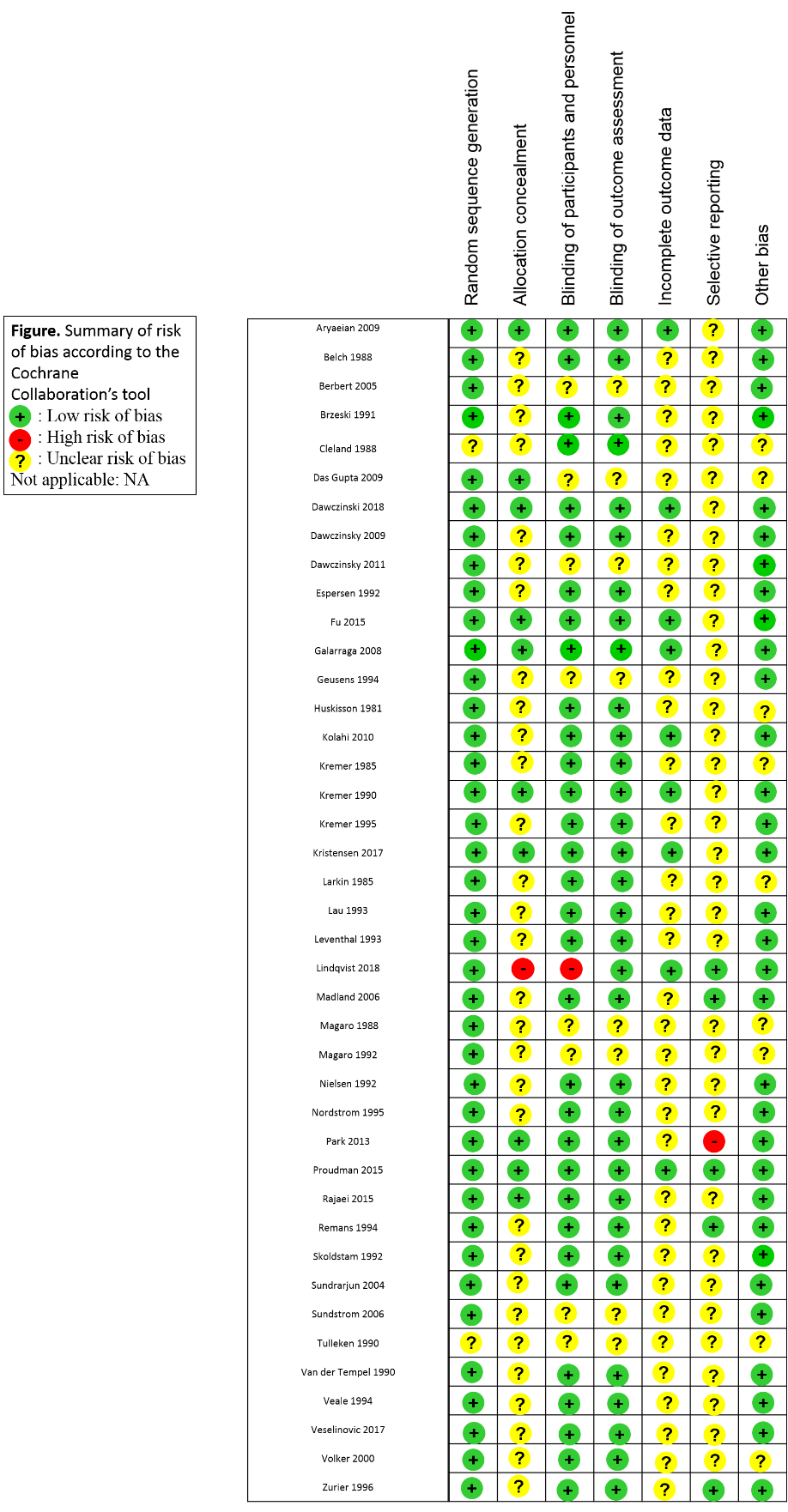
**

Supplement: Supplementary file 3 — Additional file 3. Risk of bias according to the Cochrane Collaboration Risk of Bias tool. [file 13075_2022_2781_MOESM3_ESM.docx]

**Additional file 6. Forest plot of effect of oral PUFA supplementation on tender joint count in RA**


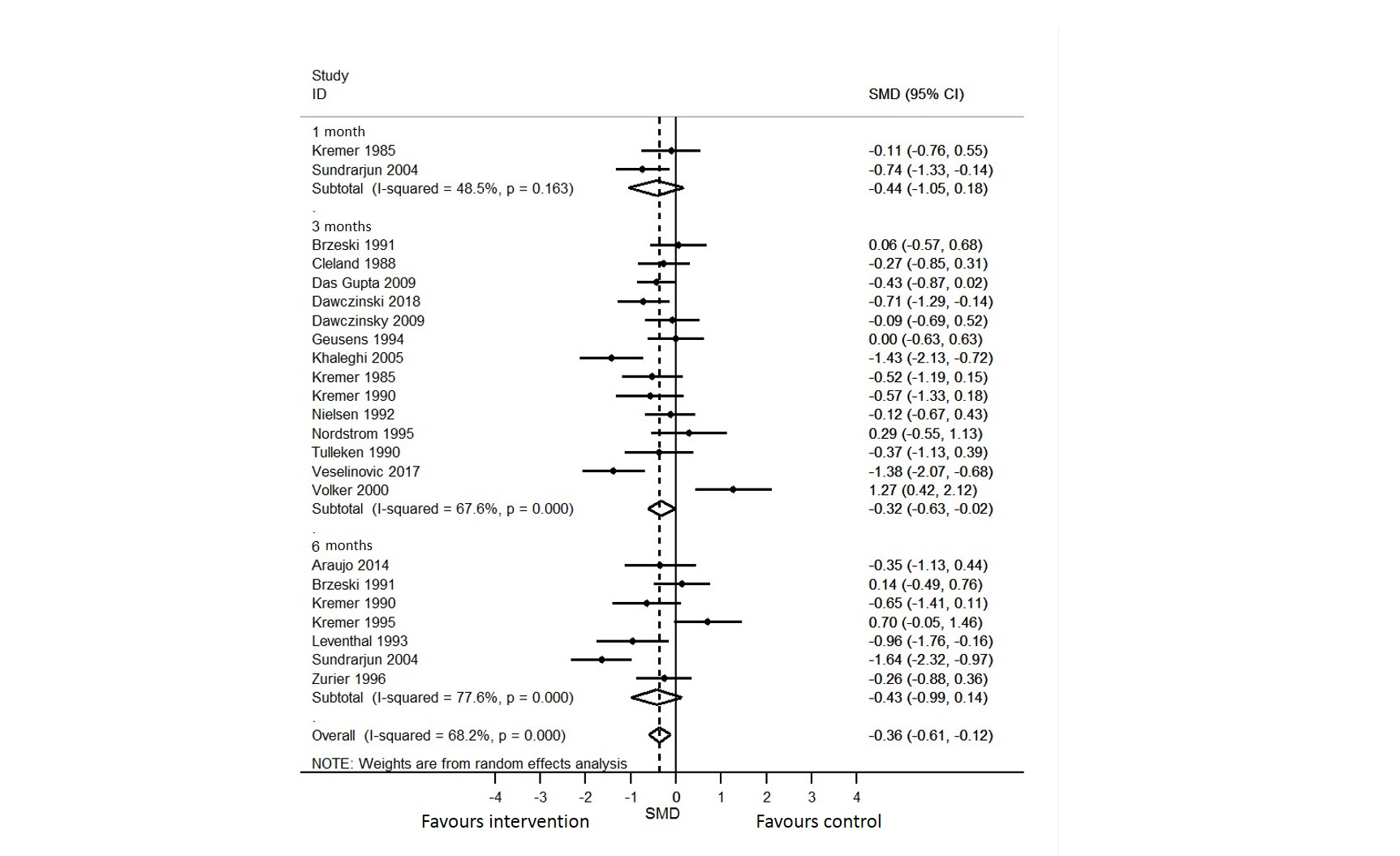

Supplement: Supplementary file 6 — Additional file 6. Forest plot of effect of oral PUFA supplementation on tender joint count in RA. [file 13075_2022_2781_MOESM6_ESM.docx]

**Additional file 11. Forest plot of effect of oral PUFA supplementation on DAS28 in RA**


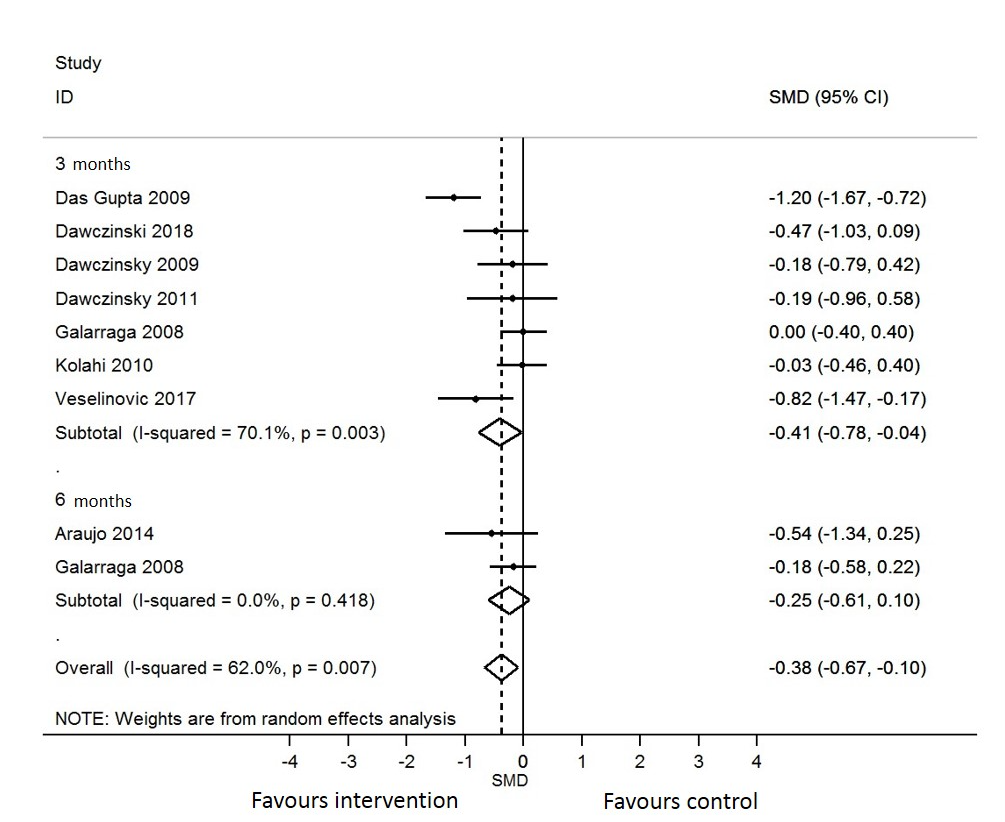

Supplement: Supplementary file 11 — Additional file 11. Forest plot of effect of oral PUFA supplementation on DAS28 in RA. [file 13075_2022_2781_MOESM11_ESM.docx]

**Additional file 12. Forest plot of effect of oral PUFA supplementation on ESR in RA**


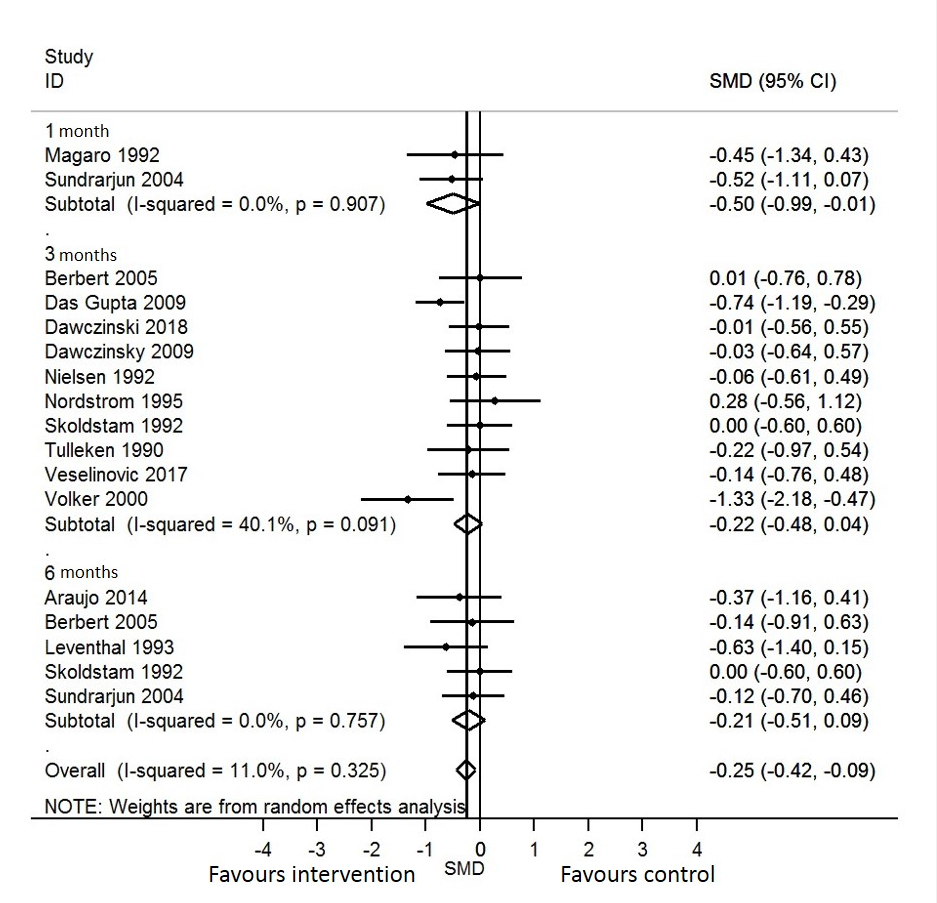

Supplement: Supplementary file 12 — Additional file 12. Forest plot of effect of oral PUFA supplementation on ESR in RA. [file 13075_2022_2781_MOESM12_ESM.docx]

**Additional file 13. Forest plot of effect of oral PUFA supplementation on CRP level in RA**


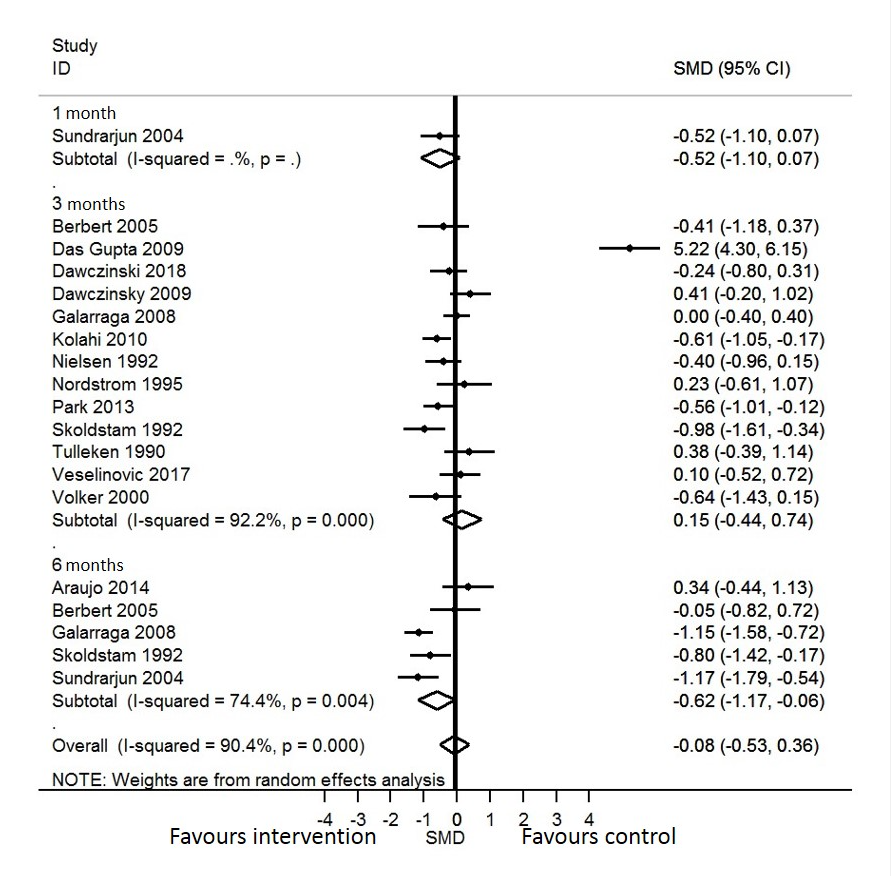

Supplement: Supplementary file 13 — Additional file 13. Forest plot of effect of oral PUFA supplementation on CRP level in RA. [file 13075_2022_2781_MOESM13_ESM.docx]

**Additional file 14. Forest plot of effect of oral PUFA supplementation on HAQ score in RA**


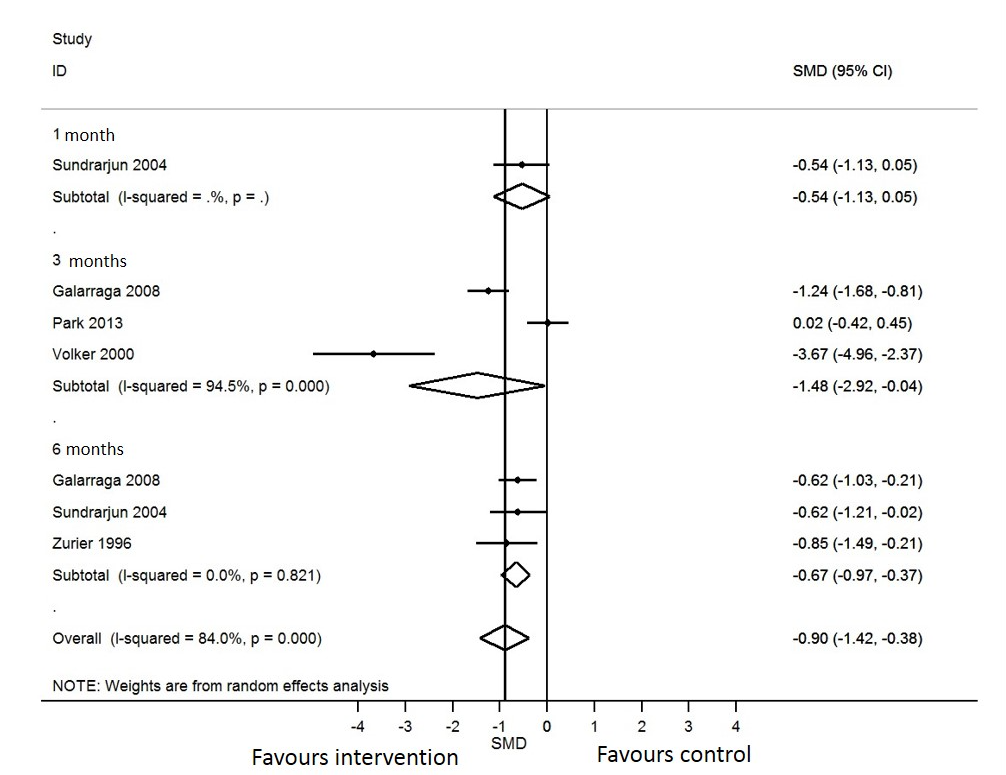

Supplement: Supplementary file 14 — Additional file 14. Forest plot of effect of oral PUFA supplementation on HAQ score in RA. [file 13075_2022_2781_MOESM14_ESM.docx]

**
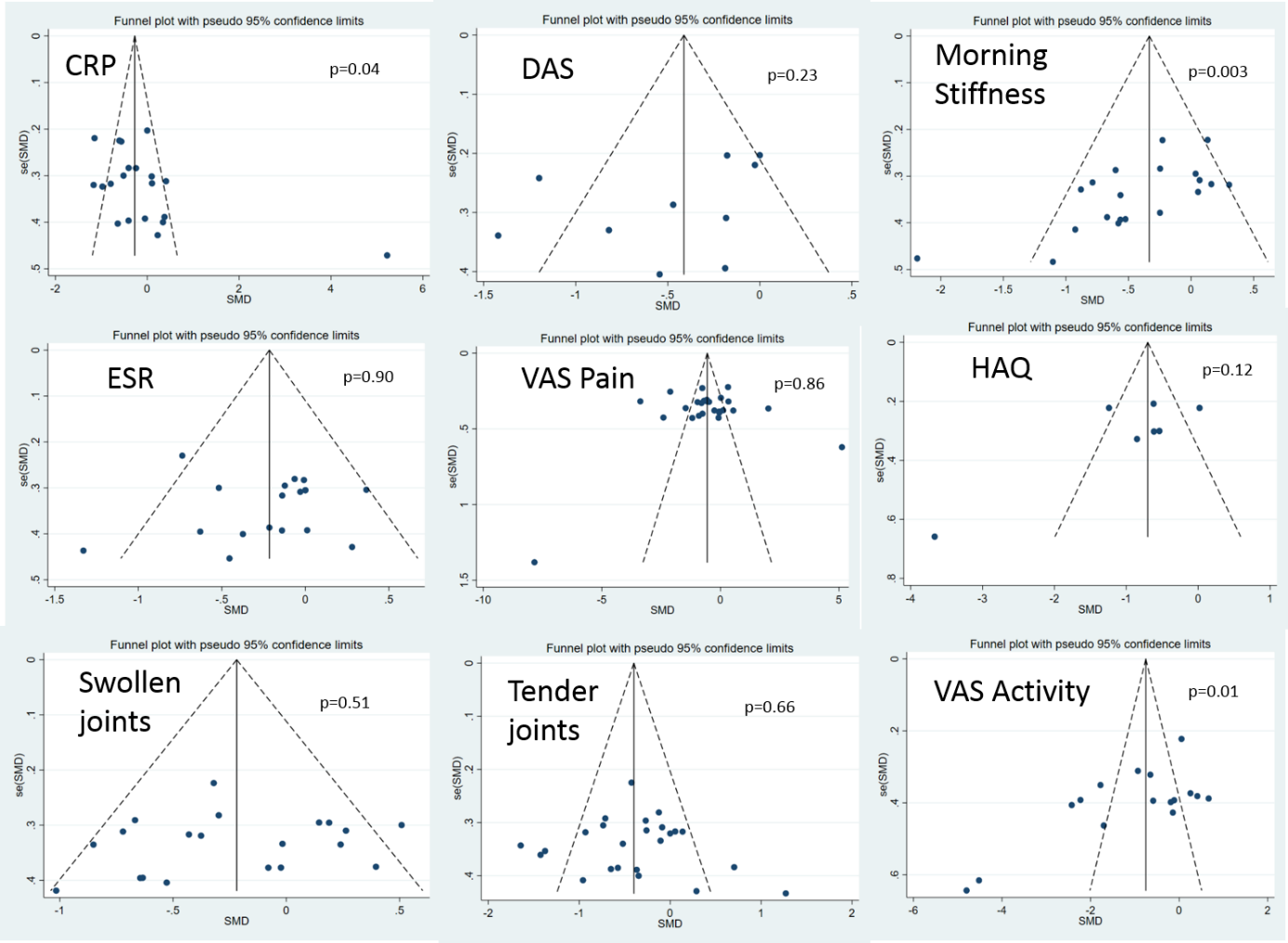
**

**Additional file 16*.* Funnel plots of publication bias**

Supplement: Supplementary file 16 — Additional file 16. Funnel plots of publication bias. [file 13075_2022_2781_MOESM16_ESM.docx]
