# Supplementary material for: Impact of type and dose of oral polyunsaturated fatty acid supplementation on disease activity in inflammatory rheumatic diseases: a systematic literature review and meta-analysis
Source: Arthritis Res Ther. 2022 May 7;24:100. doi: 10.1186/s13075-022-02781-2 (PMC9077862; doi:10.1186/s13075-022-02781-2)
Supplement: Supplementary file 4 — Additional file 4. Population baseline characteristics of studies included in the meta-analysis. [file 13075_2022_2781_MOESM4_ESM.docx]

**Additional file 4. Population baseline characteristics of studies included in the meta-analysis**

| Study | Country | Disease (classification) | Intervention group | | | | | | | Control group | | | |
| --- | --- | --- | --- | --- | --- | --- | --- | --- | --- | --- | --- | --- | --- |
|  |  |  | Age  (years) | | Disease duration (years) | | DAS28 | | Treatment status | Age  (years) | Disease duration (years) | DAS28 | Treatment status |
| Araujo 2014 (20) | Portugal | RA (ACR 87) | NR | | NR | | 3.65 (1.26) | | NR | NR | NR | 3.58 (0.91) | NR |
| Aryaeian 2009 (69) | Iran | RA (ACR 87) | 46.2 (13.1) | | 9.9 (8.4) | | 4.63 (1.26) | | NR | 47.9 (11.14) | 8.9 (8.65) | 4.35 (0.95) | NR |
| Berbert 2005 (32) | Brazil | RA (ACR 91) | 51.0 (13) | | 15 (12) | | NR | | NSAID + SAARD | 48.0 (10) | 15 (11) | NR | NSAID + SAARD |
| Brzeski 1991 (33) | Britain | RA | 60.0 | | 8 | | NR | | NSAID + DMARD  No prednisone | 61.0 | 8 | NR | NSAID and DMARD  No prednisone |
| Cleland 1988 (34) | Australia | RA (ACR 87) | 51.0 | | 8 | | NR | | Conventionnal therapies | 50.0 | 8.5 | NR | Conventionnal therapies |
| Das Gupta 2009 (24) | Bangladesh | RA (ACR 87) | 44.7 (57.7) | | NR | | 7.2 (0.4) | | Indomethacin | 49.9 (8.2) | NR | 7.3 (0.4) | Indomethacin |
| Dawczinsky 2009* (35) | Germany | RA (ACR 87) | 57.9 (10.8) | | NR | | 4.45 (1.05) | | NSAID or prednisone (<15mg/d)  +DMARD | 57.9 (10.8) | NR | 4.18 (1.11) | NSAID or prednisone (<15mg/d)  +DMARD |
| Dawczinsky 2011 (36) | Germany | RA (ACR 87) + PsA (ESSG) | Age: 56 (13)  Disease duration: NR  DAS28: 4.5 (0.15)  Treatment status : NSAID or prednisone (<15mg/d) +DMARD | | | | | | | | | | |
| Dawczinski 2018* (37) | Germany | RA (ACR 2010) | 61.3 (12.8) | | NR | | 4.3 (0.8) | | NSAID or prednisone (<15mg/d)  +DMARD | 61.3 (12.8) | NR | 4.3 (0.8) | NSAID or prednisone (<15mg/d)  +DMARD |
| Galarraga 2008 (38) | Britain | RA (ACR 87) | 58.0 | | 13 (1.26) | | 4.5 (0.15) | | NSAID  prednisone <7.5 mg/d | 61.0 | 13 (1.4) | 4.5 (0.16) | NSAID  prednisone <7.5 mg/d |
| Geusens 1994 (23) | Belgium | RA (ACR 87) | 57 (2) | 59 (2) | 9.9 (1.2) | 10 (2.2) | NR | | NSAID + DMARD | 56.0 (2) | 10.2 (1.2) | NR | NSAID + DMARD |
| Khaleghi 2005 (21) | Iran | RA (ACR 87) | 43.0 (11.5) | | 9.4 | | NR | | Conventionnal therapies | 45.0 (13) | 7.6 | NR | Conventionnal therapies |
| Kolahi 2010 (39) | Iran | RA (ARA) | 50.0 | | 4.7 | | 2.42 | | NSAID or prednisone + DMARD | 50.0 | 4.5 | 2.98 | NSAID or prednisone + DMARD |
| Kremer 1985 (22) | USA | RA (ACR 58) | 55.2 | | NR | | NR | | Hydroxycholoroquine, prednisone, gold | 56.5 | NR | NR | Hydroxycholoroquine, prednisone, gold |
| Kremer 1990 (40) | USA | RA (ACR 58) | 59.0 | 58.0 | 12.8 | 15 | NR | | NSAID + SAARD | 58.0 | 13.5 | NR | NSAID + SAARD |
| Kremer 1995 (41) | USA | RA (ACR 87) | 58.0 | | 11 | | NR | | Diclofenac or prednisone + DMARD | 57.0 | 10 | NR | Diclofenac or prednisone + DMARD |
| Kristensen 2017 (42) | Denmark | PsA (CASPAR) | 53.2 (11.4) | | NR | | 2.5 (0.9) | | NSAID+Paracetamol  +DMARD | 50.7 (11.5) | NR | 2.7 (0.9) | NSAID+Paracetamol  +DMARD |
| Leventhal 1993 (43) | USA | RA (ACR 87) | 58.0 (13) | | 9.8 (10.8) | | NR | | NSAID or prednisone<10 mg/d | 50.0 (16) | 8.9 (10.5) | NR | NSAID or prednisone<10 mg/d |
| Madland 2006 (44) | Norway | PsA | 56.9 (11.5) | | 11.5 | | NR | | NSAID+ DMARD | 53.0 (10.6) | 14.5 | NR | NSAID+ DMARD |
| Magaro 1992 (64) | Italy | RA (ACR 87) | NR | | NR | | NR | | Only NSAID | NR | NR | NR | Only NSAID |
| Nielsen 1992 (46) | Denmark | RA (ACR 87) | NR | | NR | | NR | | NSAID +prednisone+ SAARD | NR | NR | NR | NSAID +prednisone+ SAARD |
| Nordstrom 1995 (47) | Finland | RA (ACR 87) | 51.0 | | 9.6 | | NR | | NSAID+Prednisone  +SAARD | 53.0 | 13.8 | NR | NSAID+Prednisone  +SAARD |
| Park 2013 (70) | South Korea | RA (ACR 2010) | 49.2 (10.5) | | 9.5 (7.9) | | NR | | NSAID, prednisone or DMARD | 47.6 (8.8) | 7.3 (6) | NR | NSAID, prednisone or DMARD |
| Skoldstam 1992 (49) | Sweden | RA (ACR 87) | 58.0 | | 17 .0 | | 4.4 | | NSAID, prednisone <12.5mg/d + DMARD | 59.0 | 10.0 | 4.3 | NSAID, prednisone <12.5mg/d + DMARD |
| Sundrarjun 2004 (50) | Thailand | RA (ACR 87) | 46.2 (0.5) | | 4.4 (1.9) | | NR | | NSAID or prednisone + DMARD | 46.0 (0.5) | 4.4 (1.4) | NR | NSAID or prednisone + DMARD |
| Sundstrom 2006 (51) | Sweden | AS | 41.0 (15.3) | | 16.0 (15.2) | | 4.3 (1.3)** | | Only NSAID | 52.0 (17.8) | 18.0 (10.0) | 3.01 (2.39)** | Only NSAID |
| Tulleken 1990 (52) | Netherlands | RA (ACR 58) | 52.0 | | 18.0 | | NR | | NSAID+DMARD | 58.0 | 20.0 | NR | NSAID+DMARD |
| Veale 1994 (53) | Britain | PsA | 40.0 | | 9 | | NR | | NSAID+paracetamol | 40.0 | 11.0 | NR | NSAID+paracetamol |
| Veselinovic 2017 (54) | Serbia | RA (ACR 2010) | 54.0 (8) | 57.0 (8) | 6.6 (4) | 8.1 (2.7) | 4.99 (0.88) | 4.76 (0.85) | NSAID, prednisone <10mg/d + DMARD | 59.0 (7) | 5.2 (2.6) | 4.66 (0.8) | NSAID, prednisone <10mg/d + DMARD |
| Volker 2000 (55) | Australia | RA | 54.0 (4) | | 12.0 (3) | | NR | | NSAID+DMARD | 60.0 (3) | 15.0 (2) | NR | NSAID+DMARD |
| Zurier 1996 (56) | USA | RA (ACR 87) | 57.4 (9.98) | | 12.0 (6.1) | | NR | | NSAID+prednisone | 54.6 (13.58) | 10.3 (8.7) | NR | NSAID+prednisone |

Data are mean (SD). ACR= American College of Rheumatology; AS= ankylosis spondylitis; BASDAI= Bath Ankylosing Spondylitis Disease Activity Index; CASPAR= Classification Criteria for Psoriatic Arthritis; DAS28= Disease Activity Score in 28 joints; DMARD= disease-modifying antirheumatic drugs; ESSG= European Spondylitis Study Group; NR= not reported; NSAID= non-steroidal anti-inflammatory drug; PsA= psoriatic arthritis; RA= rheumatoid arthritis; SAARD= slow-acting anti rheumatic drug.

*: cross-over studies; ** BASDAI
