# Supplementary material for: Impact of type and dose of oral polyunsaturated fatty acid supplementation on disease activity in inflammatory rheumatic diseases: a systematic literature review and meta-analysis
Source: Arthritis Res Ther. 2022 May 7;24:100. doi: 10.1186/s13075-022-02781-2 (PMC9077862; doi:10.1186/s13075-022-02781-2)
Supplement: Supplementary file 5 — Additional file 5. Effect of PUFA supplementation compared to control on parameters of rheumatoid arthritis. [file 13075_2022_2781_MOESM5_ESM.docx]

**Additional file 5. Effect of PUFA supplementation compared to controls on parameters of rheumatoid arthritis**

| **Parameters** | **Effect of PUFA supplementation over time in RA** | | | | **I^2^** |
| --- | --- | --- | --- | --- | --- |
|  | **After 1 month of treatment** | **After 3 months of treatment** | **After 6 months of treatment** | **Overall effect** |  |
| Tender joints | N=2  n=82 (42+40)  -0.44 [-1.05, 0.18] | N=15  n=612 (308+304)  -0.37 [-0.66, -0.08] | N=7  n=238 (121+117)  -0.43 [-1.00, 0.14] | -0.39 [-0.63, -0.15]  p=0.001 | 68%R |
| Swollen joints | N=2  n=82 (42+40)  0.21 [-0.22, 0.65] | N=12  n=511 (259+252)  -0.35 [-0.57, -0.13] | N=6  n=198 (102+96)  -0.11 [-0.51, 0.30] | -0.23 [-0.42, -0.04]  p=0.02 | 43%  R |
| Morning stiffness duration | N=2  n=56 (29+27)  -0.47 [-1.60, 0.66] | N=12  n=553 (280+273)  -0.24 [-0.43, -0.06] | N=6  n=192 (100+92)  -0.77 [-1.39, -0.16] | -0.40 [-0.62, -0.18]  p<0.001 | 56%R |
| VAS pain | N=2  n=66 (33+33)  -2.82 [-12.5, 6.8] | N=14  n=641 (322+319)  -0.57 [-0.96, -0.18] | N=10  n=417 (213+204)  -0.35 [-1.45, 0.75] | -0.53 [-1.03, -0.03]  p=0.04 | 93%R |
| VAS activity | N=1  n=46 (23+23)  -1.78 [-2.46, -1.09] | N=7  n=266 (136+130)  -1.08 [-2.16, -0.009] | N=8  n=280 (143+137)  -1.04 [-1.92, -0.16] | -1.10 [-1.73, -0.48]  p=0.001 | 91%R |
| DAS28 |  | N=8  n=463 (231+232)  -0.52 [-0.92, -0.13] | N=2  n=123 (60+63)  -0.25 [-0.61, 0.11] | -0.48 [-0.80, -0.17]  p=0.003 | 71% R |
| CRP | N=1  n=46 (23+23)  -0.52 [-1.10, 0.07] | N=14  n=713 (357+356)  0.15 [-0.40, 0.69] | N=5  n=238 (118+120)  -0.62 [-1.17, -0.06] | -0.08 [-0.50, 0.35]  p=0.73 | 90%R |
| ESR | N=2  n=72 (36+36)  -0.50 [-0.99, -0.01] | N=11  n=452 (227+225)  -0.16 [-0.42, 0.10] | N=5  n=168 (83+85)  -0.21 [-0.39, -0.04] | -0.21 [-0.39, -0.04]  p=0.02 | 22% F |
| HAQ | N=1  n=46 (23+23)  -0.54 [-1.13, 0.05] | N=3  n=204 (103+101)  -1.48 [-2.92, -0.04] | N=3  n=184 (94+90)  -0.67 [-0.97, -0.37] | -0.90 [-1.42, -0.38]  p=0.001 | 84%R |

Data are standarized mean difference [95% confidence interval].

VAS= visual analog scale; DAS28= Disease Activity Score in 28 joints; ESR= erythrocyte sedimentation rate; CRP= C-reactive protein; HAQ= Health Assessment Questionnaire

N=number of studies; n=number of patients; standardized mean difference

n=Z (X+Y): Z=number of participants (X=number of patients receiving PUFAs + Y=number of controls)

R= random effects analysis; F= fixed effects analysis
