# Supplementary material for: Impact of type and dose of oral polyunsaturated fatty acid supplementation on disease activity in inflammatory rheumatic diseases: a systematic literature review and meta-analysis
Source: Arthritis Res Ther. 2022 May 7;24:100. doi: 10.1186/s13075-022-02781-2 (PMC9077862; doi:10.1186/s13075-022-02781-2)

**Additional file 10. Forest plot of effect of oral PUFA supplementation on patient-reported disease activity (VAS) in RA**


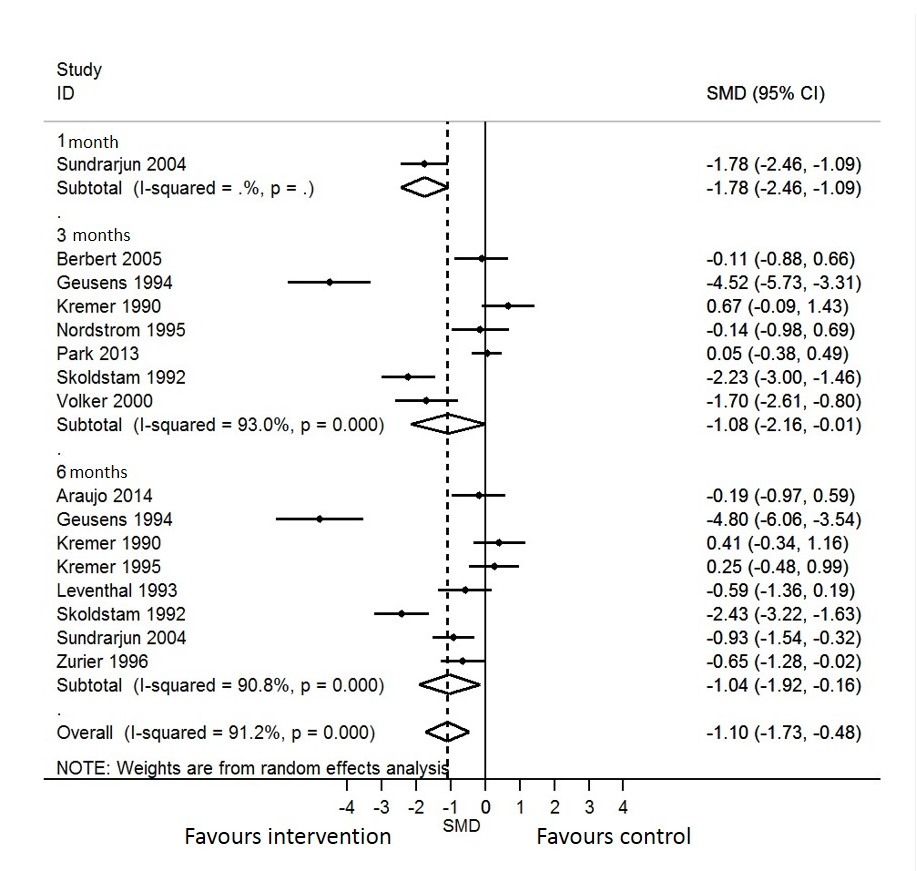

Supplement: Supplementary file 10 — Additional file 10. Forest plot of effect of oral PUFA supplementation on patient-reported disease activity (VAS) in RA. [file 13075_2022_2781_MOESM10_ESM.docx]
