# Supplementary material for: Impact of type and dose of oral polyunsaturated fatty acid supplementation on disease activity in inflammatory rheumatic diseases: a systematic literature review and meta-analysis
Source: Arthritis Res Ther. 2022 May 7;24:100. doi: 10.1186/s13075-022-02781-2 (PMC9077862; doi:10.1186/s13075-022-02781-2)
Supplement: Supplementary file 15 — Additional file 15. Effect of oral PUFA supplementation on RA disease activity by disease activity (DAS28< or > 3.2). [file 13075_2022_2781_MOESM15_ESM.docx]

**Additional file 15**. **Effect of oral PUFA supplementation on RA disease activity by disease activity (DAS28< or > 3.2).**


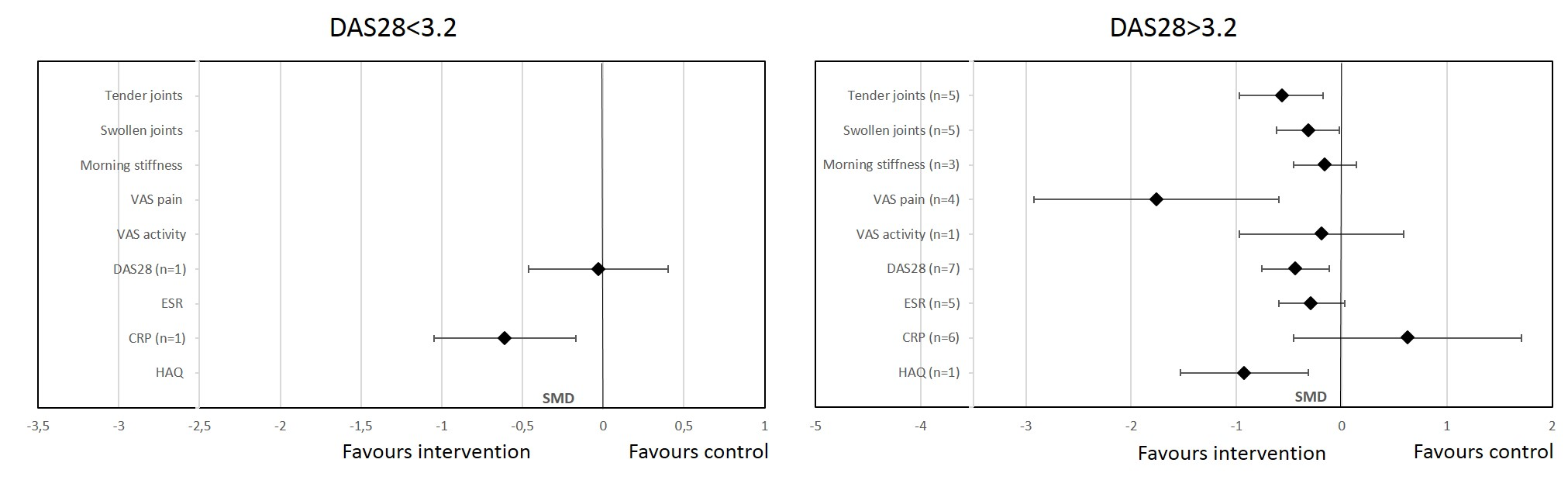


Data are standardized mean difference (SMD) (95% CI).

VAS= visual analog scale; DAS28= Disease Activity Score in 28 joints; ESR= erythrocyte sedimentation rate; CRP= C-reactive protein; HAQ= Health Assessment Questionnaire
